# Photochemical Internalization Enhanced Vaccination Is Safe, and Gives Promising Cellular Immune Responses to an HPV Peptide-Based Vaccine in a Phase I Clinical Study in Healthy Volunteers

**DOI:** 10.3389/fimmu.2020.576756

**Published:** 2021-01-08

**Authors:** Tone Otterhaug, Sylvia Janetzki, Marij J. P. Welters, Monika Håkerud, Anne Grete Nedberg, Victoria Tudor Edwards, Sanne Boekestijn, Nikki M. Loof, Pål Kristian Selbo, Hans Olivecrona, Sjoerd H. van der Burg, Anders Høgset

**Affiliations:** ^1^ PCI Biotech AS, Oslo, Norway; ^2^ ZellNet Consulting, Inc., Fort Lee, NJ, United States; ^3^ Department of Medical Oncology, Oncode Institute, Leiden University Medical Center, Leiden, Netherlands; ^4^ Department of Radiation Biology, Institute for Cancer Research, The Norwegian Radium Hospital, Oslo University Hospital, Oslo, Norway

**Keywords:** photochemical internalization, vaccine delivery, peptide vaccines, immunologic adjuvant, multifunctional T-cells, phase I study photochemical enhancement of T-cell responses

## Abstract

**Background and Aims:**

Photochemical internalization (PCI) is a technology for inducing release of endocytosed antigens into the cell cytosol *via* a light-induced process. Preclinical experiments have shown that PCI improves MHC class I antigen presentation, resulting in strongly enhanced CD8+ T-cell responses to polypeptide antigens. In PCI vaccination a mixture of the photosensitizing compound fimaporfin, vaccine antigens, and an adjuvant is administered intradermally followed by illumination of the vaccination site. This work describes an open label, phase I study in healthy volunteers, to assess the safety, tolerability, and immune response to PCI vaccination in combination with the adjuvant poly-ICLC (Hiltonol) (ClinicalTrials.gov Identifier: NCT02947854).

**Methods:**

The primary objective of the study was to assess the safety and local tolerance of PCI mediated vaccination, and to identify a safe fimaporfin dose for later clinical studies. A secondary objective was to analyze the immunological responses to the vaccination. Each subject received 3 doses of HPV16 E7 peptide antigens and two doses of Keyhole Limpet Hemocyanin (KLH) protein. A control group received Hiltonol and vaccine antigens only, whereas the PCI groups in addition received fimaporfin + light. Local and systemic adverse effects were assessed by standard criteria, and cellular and humoral immune responses were analyzed by ELISpot, flow cytometry, and ELISA assays.

**Results:**

96 healthy volunteers were vaccinated with fimaporfin doses of 0.75–50 µg. Doses below 17.5 µg were safe and tolerable, higher doses exhibited local tolerability issues in some study subjects, mainly erythema, and pain during illumination. There were few, and only mild and expected systemic adverse events. The employment of PCI increased the number of subjects exhibiting a T-cell response to the HPV peptide vaccine about 10-fold over what was achieved with the antigen/Hiltonol combination without PCI. Moreover, the use of PCI seemed to result in a more consistent and multifunctional CD8+ T-cell response. An enhancement of the humoral immune response to KLH vaccination was also observed.

**Conclusions:**

Using PCI in combination with Hiltonol for intradermal vaccination is safe at fimaporfin doses below 17.5 µg, and gives encouraging immune responses to peptide and protein based vaccination.

## Introduction

T-cell-mediated immunity is important for the control of cancer and infections by viruses, intracellular bacteria and parasites. Hence, vaccines against such diseases should be designed to induce proper T-cell responses. T-cell responses can readily be induced by vaccines based on viral vectors and nucleic acids. In contrast, subunit vaccines based on polypeptide antigens are generally good at generating antibody responses, but cellular immune responses, and especially CD8+ T-cell responses, are often inadequate. An important reason for this may be insufficient presentation of exogenously added vaccine antigens on MHC class I molecules on antigen presenting cells (APCs). Such presentation generally requires that the antigen is present in the cytosol of the APCs. Although some specialized dendritic cells has the ability to translocate antigens to the cytosol [reviewed in (1)], in most APCs the antigens stay inside intracellular vesicles in the APC with poor access to the cytosol.

Photochemical internalization (PCI) is a technology where endocytosed molecules can be released into the cell cytosol *via* a light-induced process (2). The endosomal escape induced by PCI results in enhanced access for antigens to the MHC class I presentation pathway (3, 4), as well as strongly enhanced CD8+ T-cell responses in mice (3–9). Vaccination with PCI is based on the utilization of an amphiphilic membrane-docking photosensitizing molecule (TPCS_2a_ or fimaporfin) (10) in combination with the vaccine antigen. After endocytosis, the PCI-photosensitizer and the antigen co-localizes to endosomes and lysosomes. Light-controlled activation of the photosensitizer results in reactions with molecular oxygen (O_2_) and generation of reactive oxygen species (ROS) (11). These ROS species can induce lipid peroxidation and permeabilization of the vesicle membranes, ultimately leading to the release of the endosomal content into the cytosol [reviewed in (12, 13)]. The fimaporfin photosensitizer is also used to enhance the efficacy of cytotoxic drugs, and is under clinical development for cancer therapy (14).

In addition to proper antigen presentation, the upregulation of co-stimulatory molecules and the production of cytokines are necessary signals for a proper priming of CD8+ T-cells (15). For polypeptide-based vaccination the two latter signals can be provided by immunological adjuvants inducing activation and maturation of APCs (16). Certain adjuvants can also to some degree induce cross presentation of peptide and protein antigens on MHC class I (17), but this effect is often not sufficient for a proper priming of CD8+ T-cells after vaccination. It therefore seemed logical to combine the enhanced MHC class I presentation provided by PCI with an adjuvant with a strong APC activating effect. As shown in pre-clinical experiments, combining PCI with poly(IC) based adjuvants gives a strong synergistic effect on the CD8+ T-cell response to vaccination (Selbo *et al*., manuscript in preparation). Somewhat surprisingly, in these experiments it also was found that PCI also improved helper T-cell and antibody responses

Here we present results from a phase I clinical study in healthy volunteers, showing that PCI-based peptide and protein vaccination with a poly(IC) based adjuvant is safe and results in enhanced cellular and humoral immune responses, similar to what has been observed in animal studies.

## Materials and Methods

### Study Design and Participants

This was an open label, phase I study to assess safety, tolerability, and immune response to vaccination with fimaporfin-induced PCI with antigens and adjuvant in healthy volunteers. The clinical study was done at Covance Clinical Research Unit Ltd., Leeds, UK and participants were recruited through their subject database and *via* advertisement on the Covance website and in social media. All subjects gave written informed consent and the trial was conducted in accordance with the principles of the Declaration of Helsinki and Good Clinical Practice. The study was approved in the UK by Medicines and Healthcare Products Regulatory Agency (CTA 34788/0006/001-0015) and the North East–York Research Ethics Committee (16/NE/0198). The ClinicalTrials.gov Identifier was NCT02947854.

The primary objective of the study was to assess the safety of PCI mediated vaccination. The safety endpoints were: Adverse events (graded according to the National Cancer Institute (NCI) Common Terminology Criteria for Adverse Events (CTCAE v4.03); laboratory safety evaluations; vital sign assessments; and local tolerance as assessed by pain, erythema, edema, induration, and ulceration. The secondary objective of the study was to analyze the immunological responses to PCI-mediated vaccination, with endpoints of: induction of antigen-specific T-cells measured by enzyme-linked ImmunoSpot (ELISpot) quantification of interferon-gamma (IFN-γ) releasing cells; and induction of KLH-specific antibodies.

The inclusion criteria included: Caucasian males or females, between 18 and 55 years of age, body mass index between 18.0 and 32.0 kg/m^2^, body weight between 50 and 100 kg, and evaluated to be in good health. In addition, because the HPV16 E7 peptide antigens used are known to contain HLA-A2 restricted epitopes (18), subjects had to be human leukocyte antigen A2 (HLA-A2) positive to be included in the fimaporfin dose-finding part of the study (not a criterion in the safety run-in part, see below). The exclusion criteria included: i) known previous exposure to KLH or HPV16; ii) pregnancy or breastfeeding; iii) any medication (including steroids) within 14 days of the first dose administration, that could interfere with the study procedures or compromise safety.

### Materials

The PCI photosensitizer fimaporfin [meso-tetraphenyl chlorin disulfonate (TPCS_2a_)] was obtained from PCI Biotech (Oslo, Norway). Fimaporfin was provided at 30 mg/ml in 3% polysorbate 80, 2.8% mannitol, 50 mM Tris, pH 8.5 (Amphinex formulation) and was kept light protected at 2–8°C. The adjuvant poly-ICLC (Hiltonol) is a synthetic double-stranded RNA complex of poly(IC) stabilized with poly-L-lysine polylysine and carboxymethylcellulose (19). Hiltonol 2 mg/ml was purchased from Oncovir (Washington DC, USA) and kept at 2–8°C (when aliquoted it was used within 14 days). The vaccine antigens employed in the clinical study were: Human papillomavirus type 16 (HPV16) oncoprotein E7_1-35_: MHGDTPTLHEYMLDLQPETTDLYCYEQLNDSSEEE and HPV16 E7_62-98_: DSTLRLCVQSTHVDIRTLEDLLMGTLGIVCPICSQKP both produced according to Good Manufacturing Practice by PepScan (Lelystad, Netherlands). The HPV16 E7 peptides were stored at -70°C. Keyhole Limpet Hemocyanin 1 mg/ml (KLH: Immucothel^®^) was obtained from Biosyn Arzneimittel GmbH (Fellbach, Germany) and stored at 15–20°C.

### Study Procedures

The vaccine components were mixed at the bedside within 15 min prior to injection using sterile, endotoxin-free 0.9% NaCl as diluent: 1) KLH Mix consisted of Hiltonol (50 µg), KLH (100 mg), and fimaporfin (0.75–50 µg); 2) HPV16 E7 Mix consisted of Hiltonol (50 µg), HPV16 E7_1-35_ (100 µg), HPV16 E7_62-98_ (100 µg), and fimaporfin (0.75–50 µg). After gentle mixing by hand, 150 µl was aspirated into a 0.3 ml syringe with a 30G needle (BD MicroFine^®^ 39, Becton Dickinson, Franklin Lakes, USA) and injected intradermally (ID) to the subjects. 20 h (± 4 h) later, each injection site was exposed to 652 nm red light, delivered from a CE-marked PCI Biotech laser (produced by Modulight, Tampere, Finland). When not illuminated, the injection sites were covered by dark clothing, a bandage or dressing, for 14 days after the injections.

**Figure 1:**
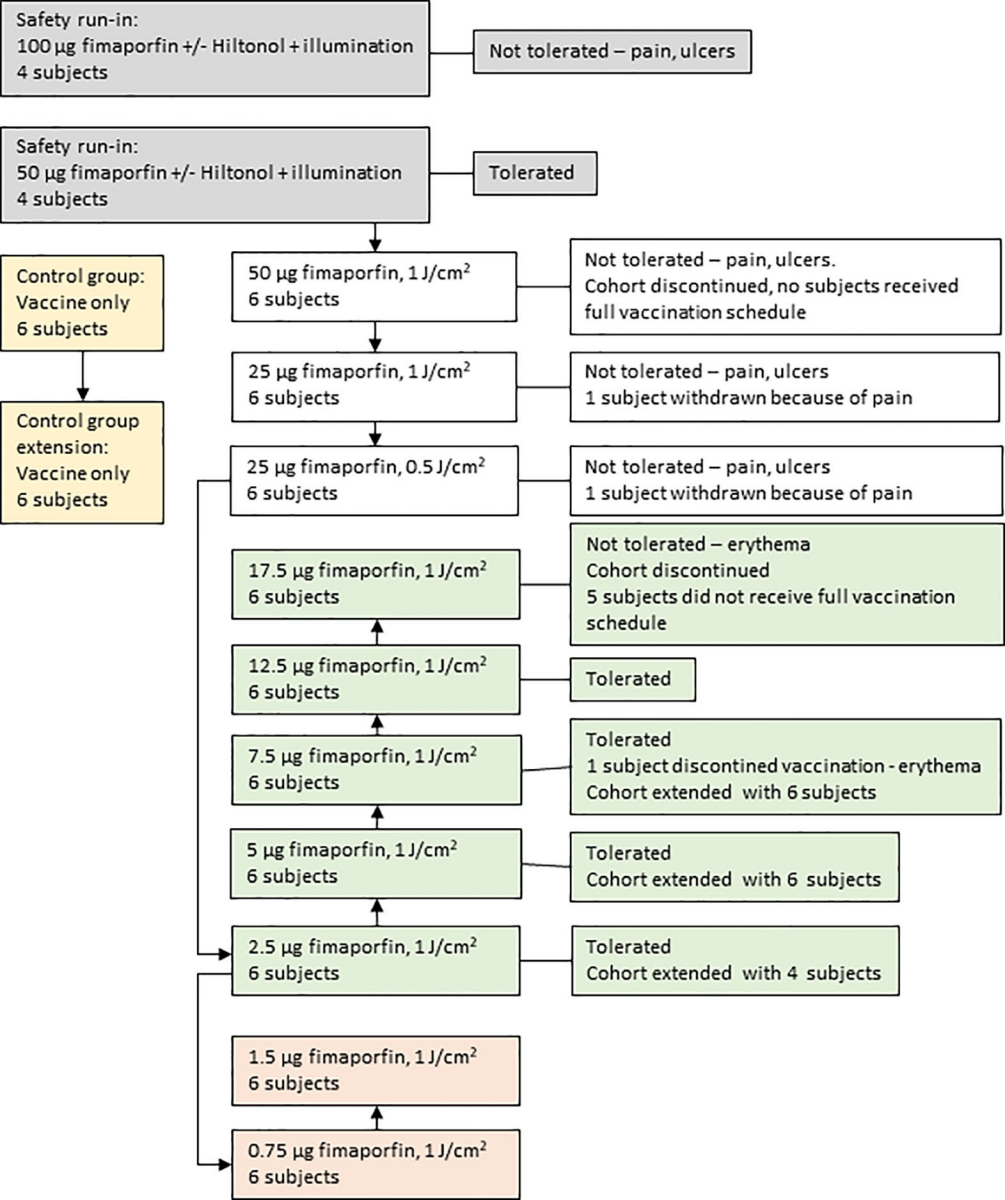
Patient disposition. The flow of study subjects through the different phases of the study is outlined. Each study subject participated in one group only. The arrows depict the temporal relationships for the consecutive treatment of the different groups. As described in detail in the main text the study started with a safety run-in part (gray boxes) where a dose of 50 µg fimaporfin was found to be tolerated. The main study (white boxes) was then started with this dose, but because of the appearance of vaccination site ulcers in some subjects the dose was reduced to 25 µg. Also this dose was not tolerated, due to the occurrence of ulcers and pain during illumination. The fimaporfin dose was therefore reduced to 2.5 µg. This dose was well tolerated, and the dose was escalated (green boxes) up to 17.5 µg, which was not tolerated due to the development of erythema of a size that exceeded the cohort stopping criteria. Due to initial promising signs of immune responses in the 2.5 µg dose cohort, also the lower doses of 0.75 and 1.5 µg fimaporfin were included in the study (light red boxes). In parallel with the first dose cohorts a control group, receiving the vaccine without fimaporfin, was performed (yellow boxes). Based on the results obtained during the study the control group and several of the fimaporfin dose groups were extended with six or four additional study subjects (as indicated on the figure), meaning the at the end of the study a total of 12 subjects had been treated in the control group and 10, 12 and 12 in the 2.5, 5 and 7.5 µg dose groups, respectively.

As is outlined in **Figure 1** the clinical study was conducted in two parts, the first part being a safety run-in to select the fimaporfin starting dose for the dose-finding part of the study. The run-in part was a sequential group, fimaporfin dose-reduction study to evaluate the safety and tolerability (pain and local reactions) of the components of PCI (fimaporfin and light), in the absence of antigen, when administered alone and in combination with Hiltonol. The fimaporfin doses tested (50 and 100 µg) were selected as they are known to be effective in mice and tolerable in minipigs (data not shown). Eight subjects were enrolled in two fimaporfin dose groups (**Table 1**); four subjects received 100 µg and four subjects received 50 µg. On day 1, each subject received a single ID dose of fimaporfin and a single ID dose of a mixture of Hiltonol and fimaporfin, at 2 separate injection sites. The illumination dose of 1 J/cm^2^ was fixed, however the irradiance regimen was different in each fimaporfin subgroup, where two subjects received the dose as 5 mW/cm^2^ for 200 s and the other two as 10 mW/cm^2^ for 100 s.

**Table 1:** Safety run-in treatment doses.

Subjects	50 µg fimaporfin				100 µg fimaporfin			
	n=2		n=2		n=2		n=2	
Injection site	1	2	1	2	1	2	1	2
Hiltonol (µg)	0	50	0	50	0	50	0	50
Irradiance (mW/cm^2^)	5	5	10	10	5	5	10	10
Illumination duration (s)	200	200	100	100	200	200	100	100

Each subject received 2 ID doses of fimaporfin, with and without the addition of Hiltonol. Four subjects received the same fimaporfin dose where two subjects received same light irradiance and illumination duration.

Subsequently, a fimaporfin dose-finding part was performed to evaluate the safety, tolerability and immune responses when PCI was combined with the vaccine antigens and adjuvant. An antigen-adjuvant control group was included to enable a comparison of effects with and without PCI. The starting dose of fimaporfin and illumination was determined based on results from the safety run-in part of the study and fimaporfin doses of 0.75–50 µg were tested with a light dose of 1 J/cm^2^. Overall, each subject received two doses of the KLH vaccine and three doses of HPV16 E7 vaccine (unless safety concerns or stopping criteria were met) given at separate, rotating injection sites in the upper arms and on the belly. The vaccine mixes were given as ID injections with 2 weeks between vaccinations.

Blood samples were drawn pre-treatment, 14 days after each vaccination and at the end of the study, 4 weeks after the last vaccination. Clinical assessments for safety as well as immunology endpoints were performed before and after each vaccination. Participants remained in the clinic from the day before dosing and for 24 h post ID dosing (control group), or 6 h post light exposure on day 2, 16, and 30, and returned for non-residential visits the following day and up to 4 weeks after last dosing.

### Safety Measures in the Study

For safety reasons a sequential-group, sentinel dosing design was chosen for the dose-finding part of the study, as the impact of antigens and adjuvant in the presence of the photosensitizer fimaporfin and illumination was unknown. This part was conducted with two sentinel subjects, dosing cohorts of 1, 1 and 4 subjects on different days. Available safety data from the first sentinel subject ≥ 40 h post light dosing was evaluated by the investigator before dosing started for the second sentinel subject, and available safety data from the second sentinel (≥ 40 h post light dosing) was evaluated before dosing started for the remaining four subjects in the dose group. Similar evaluations of available safety data were done prior to the second and third dosing occasions for the second sentinel subject and the remaining four subjects within each dose group.

Prior to the second and third dosing of each individual, available safety data from the previous dosing in the individual was evaluated. If there were safety concerns in individual subjects, subsequent doses of one or both antigen/adjuvant combinations were not given to that particular subject. Subjects were withdrawn from dosing if they experienced a serious adverse event (SAE) or had clinical signs of hypersensitivity reaction or cytokine release syndrome grade 3 or above considered to be possibly related to study treatment, or if they had grade 3 local administration site reactions or the presence of local administration site ulceration [as defined by the CTCAE criteria (Version 4.03 or later)].

Between each dose cohort, a Safety Review Committee (SRC) consisting of a minimum of the investigator, a sponsor representative, an independent immunologist, and a medical monitor, reviewed safety and tolerability data from a minimum of four subjects in the last dose group (up to at least 40 h after second dosing). An interim safety report, summarising results from all required safety assessments, was provided, and any clinically significant results were discussed before next dose cohort. Separate dose escalation decisions were made for each antigen. A minimum of 7 days separated the dosing of the last subject receiving their second dose in one group and dosing of the first subject in the next group.

Dose escalation would stop, or a lower dose be selected if ≥ 1 grade 3, or ≥ 2 grade 2, AEs of a similar nature within a dose group was considered related to the study drug. In addition, if ≥ 2 subjects in a group had grade 3 local administration site reactions (as defined above), presence of local administration site ulceration, or were not able to tolerate the complete light application, the dose would not be escalated. When the stopping criteria for a dose cohort were met, all subjects in that cohort discontinued further treatment. Doses where the stopping criteria were not met were regarded as safe and tolerable.

### Safety Assessments

Safety monitoring included vital signs and clinical laboratory evaluations, physical examinations and 12-lead ECGs. Any adverse event (AE) and remedial actions required were recorded, and any clinically significant abnormalities identified during the course of the study were followed up until they returned to normal or could be clinically explained. Local tolerability at the dosing sites was assessed prior to and after each ID injection and light application (2, 6, 24, and 48 h post ID and light dosing), and involved evaluation of pain, erythema, edema, induration, and ulceration following individual scales for each measure. Local tolerability events of grade 3 included: *Pain*: Prevents daily activity or necessitated repeated use of narcotic pain reliever; *Erythema*: More than 100 mm; *Edema*: More than 100 mm; *Induration*: height: >1 mm. Local tolerability ratings of ≥ Grade 3 and/or the presence of ulceration were recorded as adverse events. Assessment of pain intensity at the injection site before, during, and after light exposure was assessed using a visual analogue scale (VAS) from 0 to 100 mm.

### Immune Monitoring Analysis

#### Peripheral Blood Mononuclear Cells Isolation and Storage

Blood was collected in sodium heparin tubes from subjects prior to the vaccination (day 1) and post-vaccination on days 15, 29, 43, and 56. Peripheral blood mononuclear cells (PBMCs) for immune monitoring were isolated within 8 h of venipuncture by standard density gradient centrifugation using Histopaque (Sigma-Aldrich, St. Louis, USA). PBMCs were cryopreserved in Cell recovery (ThermoFisher, Waltham, USA) or CTL ABC (CTL, Shaker Heights, USA) freezing medium using Mr. Frosty (Sigma-Aldrich, St. Louis, USA). Cells were initially frozen at -80°C and transferred to liquid N_2_ within 18–48 h.

#### ELISpot Analysis

To assess cellular immunity, an interferon-γ (IFN-γ) ELISpot assay was done to determine responses to HPV16 E7 and KLH. Cryopreserved PBMC samples were thawed in a 37°C water bath until a small piece of ice remained. The PBMCs were then immediately transferred to a 15 ml Falcon tube, and 10 ml (37°C) AIM-V medium (Thermo Fisher Scientific, Waltham, USA) containing 0.5 U/ml benzonase nuclease (Sigma-Aldrich, St. Louis, USA) was added drop-wise. Cells were centrifuged 10 min at 450 × g at room temperature (RT) and re-suspended in 10 ml AIM-V (37°C) (without benzonase). After a new centrifugation, cells were re-suspended in AIM-V (37°C) at 2x10^6^ cells/ml, split into 5 ml aliquots in 50 ml tubes (with the cap not completely closed to enable CO_2_ exchange) and maintained overnight (20–24 h) in a cell incubator with a humidified atmosphere containing 5% CO_2_ at 37°C (20, 21). Counting of viable cells was performed using trypan blue staining.

For the IFN-γ ELISpot assay the Human IFN-γ ELISpot^PLUS^ (ALP) kit (3420-4ALP-10, Mabtech, Nacka Strand, Sweden) was used according to the manufacturer’s instructions. Shortly, the overnight rested cells were resuspended, and an aliquot was removed for cell counting. Cells were centrifuged at 450 g and re-suspended in AIM-V medium (37°C) at 2x10^6^ viable cells/ml. Then, 100 µl of each sample was added to the wells in the precoated ELISpot plate, 100 µl of AIM-V (37°C) containing concentrated stimulants was added for triplicate testing per condition and the plates were incubated for 20–22 h at 37°C. The HPV16 E7 peptides (the same as used for the vaccination) were dissolved in DMSO and added to the cell samples for a final concentration of 10 µg/ml, with a final DMSO concentration of 1.2%.

KLH was reconstituted in sterile solvent supplied in the kit and added to cell samples for a final concentration of 100 µg/ml. Concanavalin A (1 µg/ml final; Sigma-Aldrich, St. Louis, USA) and CEFx peptides (1 µg/ml final) (JPT Peptide Technologies Berlin, Germany) were added to the samples in separate wells as positive controls, while cells incubated with medium only served as a negative control. Each batch of tested subjects (typically 3 subjects at a time) was accompanied by testing the same PBMCs from a healthy donor (reference sample) against medium, CEFx, and ConA. Testing results of this reference control revealed robust and similar antigen and mitogen responses at all testing dates. The next day cells were removed from the plate and the wells were washed 5 times with PBS before the addition of 100 µl 1 µg/ml biotinylated monoclonal Antibody 7-B6-1 working solution. Following incubation at RT for 2 h and 5 washes, the plates were incubated with Streptavidin-ALP for 1 h before washing again. The plates were developed until spots emerged (usually around 3 min) using the substrate BCIP/NBT dissolved in deionized water. Plates were left to dry in the dark and spots were enumerated at ZellNet Consulting Inc. (Fort Lee, USA) with a KS ELISpot reader (Zeiss, Thornwood, USA), using Software version KS ELISpot 4.9.16 following the International harmonization guidelines for ELISpot plate evaluation (22).

The Limit of Detection (LOD) in the ELISpot assay was determined from the actual trial subjects tested. Using the spot counts in the negative control wells from all the samples, the median spot count was calculated to be 5. Therefore, using a signal: noise ratio of 3:1 for defining the LOD, 15 spots was considered the LOD for this study. This LOD was used as the basis for calculations of response rates.

Two definitions for an antigen-specific response were used: i) A statistical Distribution-Free Resampling (DFR) test in the setting where triplicate wells were available for all antigens (23). The DFR test provides two results: I. for any statistical difference [DFR(eq) testing results, less stringent], and II. for statistical differences which are at least 2-fold above the negative control [DFR(2x), more stringent testing] ii) An empirical rule of 2-fold or greater difference between the mean antigen spot counts compared to the mean negative control spot counts. For both tests, the average antigen spot count had to be greater than or equal to the global LOD of 15 spots to be considered a response.

The response to treatment was defined with the following rules: i) If there was no pre-existing response measured on day 1), but there was a response, measured at any of the following time points ii) If there was a pre-existing response, a response measured at any of the following time points had to be at least 2x as high as the response measured on day 1.

#### Flow Cytometry Analyses

To assess functional responses of CD4+ and CD8+ T-cells in response to stimulation with HPV16 E7 peptide pools, flow cytometry analysis was performed. Thawed PBMC samples were resuspended in Iscove’s Modified Dulbecco’s Medium (IMDM, Lonza, Basel, Switzerland), supplemented with 2mM L-glutamine, 100 U/ml penicillin, 100 µg/ml streptomycin (all from Life Technologies, Waltham, USA), and 10% human serum albumin (Albuman^®^; Sanquin Plasma Products BVAmsterdam, Netherlands) and plated in 6-wells plates (days 29, 43, and 57 were separately put into culture). The cells were stimulated with peptide pools consisting of HPV16 E7 1-35 (35-mer) + E7 61-82 (22-mer) + E7 64-98 (35-mer). The next day T-cell growth factor (TCGF; ZeptoMetrix, Buffalo NY, USA) and Interleukin-15 (IL-15; Peprotech, London, UK) was added to the bulk cultures as described previously (24, 25). The cells were cultured for 10 days (only medium with Albumin was added when required, mostly on day 7). Thawed PBMC samples (1–4 x 10^6^ cells/ml) from the corresponding day 15 samples were used for monocyte adherence in 48-wells plates (0.5 ml/well) to be used as APCs. These monocytes were cultured in X-vivo 15 medium (Lonza, Basel, Switzerland) and incubated with 800 IU/ml Granulocyte-macrophage colony stimulating factor (GM-CSF; Invitrogen, Waltham, USA) for 3 days and subsequently loaded overnight separately with the following peptide pools: i) HPV16 E7 1-35 (10-mer peptides): 1-10, 2-11, 5-14, 7-16, 10-19, 11-20, 15-24, 20-29, 26-35; ii) HPV16 E7 62-98 (10-mer peptides): 62-71, 63-72, 70-79, 73-82, 77-86, 78-87, 80-89, 82-91, 85-94, 89-98; iii) HPV16 E7 (22-mer peptides): 1-22, 11-32, 21-42, 61-82, 71-92, 77-98. Monocytes loaded with Staphylococcus enterotoxin B (SEB, Sigma) served as a positive control, and monocytes in X-vivo 15 medium only as a negative control. The 10-days cultured PBMCs were harvested, resuspended in IMDM with Albumin (2 x 10^6^ cells/ml) and added to the peptide loaded monocytes (0.5 ml/well). Brefeldin A was added after 1 h (final concentration 10 µg/ml) to prevent cytokine secretion, and the cells were after overnight stimulation harvested and subjected to intracellular cytokine staining (24, 25). Staining was done for the following markers: T-cell markers CD3, CD4, and CD8; cytokines IFN-γ, TNF-α and IL-2; and activation markers CD154 and CD137. A time-gate was set to exclude any regions of sheath flow fluctuations that could result in false-positive or false-negative events (gating tree shown in **Supplementary Figure 1**). To determine whether samples were reactive to HPV16 E7 (or the positive control SEB), measured T-cell responses were considered positive if they consisted of ≥10 events within the gate, and their frequency was at least twice that in the matched negative control (medium only). Reported results are background-subtracted except if noted otherwise.

#### KLH IgG Antibody Analysis

Serum samples collected from subjects before vaccination, 14 days after each vaccination and at the end of the study (6 weeks after last KLH vaccination) were analyzed for anti-KLH IgG using an ELISA assay (Alpha Diagnostics, San Antonio, USA). The ELISAs were performed with dilutions of positive and naïve anti-KLH IgG human serum samples and the subject samples were tested in 5 dilutions. The ELISA plate was read using a Spectramax 340PC plate reader at 450 nm. The anti-KLH IgG in a sample was determined using an antibody cut titration method. The concentration of each sample was calculated, and the average of diluted samples was determined.

## Results

### Subject Disposition

As outlined in **Figure 1**, In total 96 healthy volunteers participated in this study; 8 in the safety run-in part and 88 in the fimaporfin dose-finding part of the study. In this dose-finding part, 12 of the 88 subjects were enrolled in the control group and 76 subjects were treated with different doses of fimaporfin (**Figure 1** and **Table 2**). The 8 subjects in the safety run-in part received fimaporfin alone or in combination with Hiltonol as shown in **Table 1**. In the 100 µg fimaporfin dose group, four adverse events of grade 1 local ulceration in three patients were reported, in addition to injection site paresthesia (one event) and extravasation (one event) all being suspected as related to study treatment (**Table 3**). In the 50 µg group, no ulcerations or other prominent local reactions were observed, and 50 µg was therefore selected as the starting dose for the fimaporfin dose-finding part of the study. There were no apparent differences in safety events between subjects receiving illumination at 5 mW/cm^2^ and subjects being illuminated with 10 mW/cm^2^; and an irradiance of 5 mW/cm^2^ for 200 s, giving an illumination dose of 1 J/cm^2^, was chosen for the dose-finding part of the study. Although there were few patients in this safety run-in part of the study, the results indicated that the local reactions observed were mainly due to the photochemical treatment with fimaporfin, since there was no clear difference between the injection sites with or without Hiltonol.

**Table 2:** Baseline characteristics and dose groups assignment of subjects in the fimaporfin dose-finding part.

Dose group		Control	Low	Intermediate	High	Non-tolerated	Overall	Safety run-in
		(N=12)	(N=46)	(N=6)	(N=6)	(N=18)	(N=88)	(N=8)
Fimaporfin dose		0 μg	0.75– 7.5 μg	12.5 μg	17.5 μg	25–50 μg	0– 50 μg	50– 100 µg
Age, years (median, range)		33 (19–55)	34–42 (19–53)	44 (21–52)	44 (25–55)	30–47 (19–55)	36 (19–55)	45 (21–55)
Sex	Male	9 (75.0%)	37 (80.4%)	5 (83.3%)	4 (66.7%)	12 (66.7%)	67 (76.1%)	4 (50.0%)
	Female	3 (25.0%)	9 (19.6%)	1 (16.7%)	2 (33.3%)	6 (33.3%)	21 (23.9%)	4 (50.0%)
Race	White	12 (100.0%)	46 (100.0%)	6 (100.0%)	6 (100.0%)	18 (100.0%)	88 (100.0%)	8 (100.0%)
Ethnicity	Not Hispanic or Latino	11 (91.7%)	46 (100.0%)	6 (100.0%)	6 (100.0%)	17 (94.4%)	86 (97.7%)	8 (100.0%)
	Hispanic or Latino	1 (8.3%)	0 (0.0%)	0 (0.0%)	0 (0.0%)	1 (5.6%)	2 (2.3%)	0 (0.0%)

Median (range) or number of subject (with percentage) are given for the different characteristics.

**Table 3:** Any Related TEAEs reported in Safety Run-In part.

Dose group	50 μg fimaporfin (N=4)		100 μg fimaporfin(N=4)	
Preferred Term	Mild	Moderate	Mild	Moderate
**Any related TEAE**	–	1 (25.0%) [1]	4 (100.0%) [6]	–
**Local TEAE**	–	–	4 (100.0%) [6]	–
IS ulcer	–	–	3 (75.0%) [4]	–
IS extravasation	–	–	1 (25.0%) [1]	–
IS paresthesia	–	–	1 (25.0%) [1]	–
**Systemic TEAE**	–	1 (25.0%) [1]	–	–
Syncope	–	1 (25.0%) [1]	–	–

AEs were considered to be local symptoms were anywhere the MedDRA Preferred Term contained the term ‘Injection site’. For Related to Study Treatment this counts all TEAE that have been recorded as having a suspected relationship to study treatment with and/or without red light. N, Number of subjects studied; (), Percentage of subjects with adverse events; [], Number of adverse events. Events were coded using MedDRA (Version 19.0).

**Figure 2:**
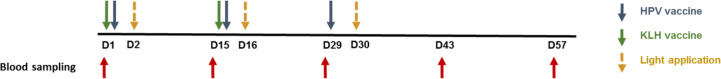
Treatment and blood sampling schedule. Subjects could receive three doses of HPV vaccine, and two doses of KLH vaccine given at 2 weeks intervals. Blood samples were collected pre- and post-vaccination at the time points indicated by red arrows.

As shown in **Figure 1** the subjects enrolled into the fimaporfin dose-finding part were divided over 10 cohorts, each consisting of 6–12 healthy volunteers, who received fimaporfin doses from 0.75 µg to 50 µg. The control group (n=12) received Hiltonol and vaccine antigens only, whereas the fimaporfin groups received PCI (fimaporfin + light) in addition to Hiltonol and vaccines. All groups received the same dose of Hiltonol and vaccine antigens, and up to five injections (**Figure 2**).

### Safety and Tolerability of PCI Mediated Vaccination

ID vaccination with PCI was safe and well tolerated (**Table 4**), and no SAEs were reported in the study. Overall, 27 of the 88 subjects (30.7%) in the dose-finding part reported 53 treatment emergent AEs (TEAEs), of which 34 were local symptoms and 19 were systemic reactions. The majority of the reported events were classified as mild, but 4 subjects in the highest dose groups (25 µg and 50 µg fimaporfin) reported five moderate TEAEs that were classified as related to the study treatment; four of these events were local reactions at the injection site. Systemic AEs related to study treatment were all mild except 1 moderate event of procedural site reaction (25 µg). Only 2 of the reported systemic symptoms (headache and procedural reaction) were reported by ≥ 2 subjects. There were 13 more different systemic AEs, each one reported by one subject, and only at one time-point. All laboratory abnormalities were classified as not related to the study intervention.

**Table 4:** Any TEAEs related to study treatment reported by ≥ 2 subjects.

Dose group	Control (N=12)		Low (N=46)		Intermediate (N=6)		High (N=6)		Non-tolerated (N=18)		Overall (N=88)	
Fimaporfin dose	0 μg		0.75–7.5 μg		12.5 μg		17.5 μg		25–50 μg		0–50 μg	
Preferred Term	Mild	Moderate	Mild	Moderate	Mild	Moderate	Mild	Moderate	Mild	Moderate	Mild	Moderate
**Any related TEAE**	4 (33%) [4]	–	6 (13%) [7]	–	1 (17%) [1]	–	5 (83%) [13]	–	9 (50%) [23]	4 (22%) [5]	25 (28%) [48]	4 (5%) [5]
**Local TEAE**	3 (25%) [3]	–	5 (11%) [6]	–	1 (17%) [1]	–	5 (83%) [12]	–	6 (33%) [8]	3 (17%) [4]	20 (23%) [30]	3 (3%) [4]
IS vesicles	—	–	2 (4%) [2]	–	–	–	3 (50%) [7]	–	2 (11%) [2]	–	7 (8.%) [11]	–
IS pruritus	3 (25%) [3]	–	2 (4%) [2]	–	–	–	–	–	2 (11%) [4]	–	7 (8.%) [9]	–
IS pain	–	–	–	–	1 (17%) [1]	–	1 (17%) [1]	–	1 (6%) [1]	1 (6%) [1]	3 (3%) [3]	1 (1%) [1]
IS ulcer	–	–	–	–	–	–	–	–	1 (6%) [1]	2 (11%) [3]	1 (1%) [1]	2 (2%) [3]
IS reaction	–	–	1 (2%) [2]	–	–	–	1 (17%) [1]	–	–	–	2 (2%) [3]	–
IS erythema	–	–	—	–	–	–	2 (33%) [2]	–	–	–	2 (2%) [2]	–
IS scab	–	–	—	–	–	–	1 (17%) [1]	–	–	–	1 (1%) [1]	–
**Systemic TEAE**	1 (8%) [1]	–	1 (2%) [1]	–	–	–	1 (17%) [1]	–	8 (44%) [15]	1 (6%) [1]	11 (13%) [18]	1 (1%) [1]
Headache	1 (8%) [1]	–	–	–	–	–	–	–	3 (17%) [3]	–	4 (5%) [4]	–
Procedural site reaction	–	–	–	–	–	–	1 (17%) [1]	–	–	1 (6%) [1]	1 (1%) [1]	1 (1%) [1]

AEs considered to be local symptoms were anywhere the MedDRA Preferred Term contained the term Injection site (IS)’. For Related to Study Treatment this counts all TEAEs that have been recorded as having a suspected relationship to study treatment with and/or without red light. N, Number of subjects studied; (), Percentage of subjects with adverse events; [], Number of adverse events. Events were coded using MedDRA (Version 19.0).

**Figure 3:**
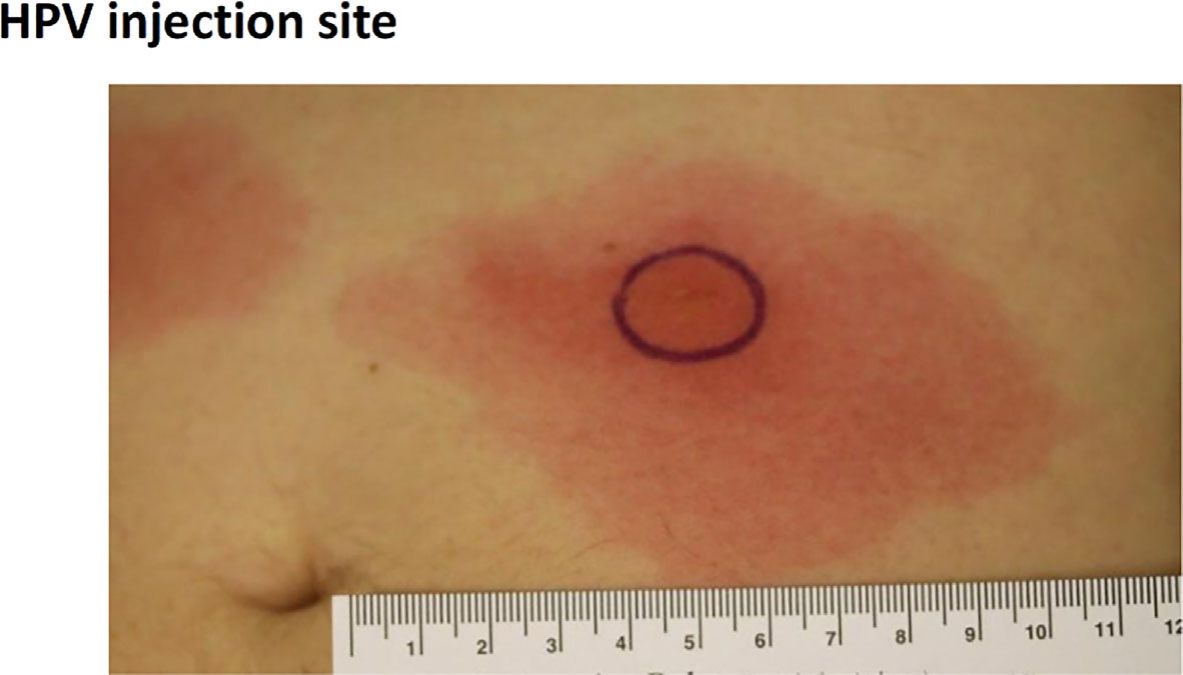
Development of vaccination site erythema induced discontinuation of treatment in the 17.5 µg dose cohort. The figure shows an example of erythema > 10 cm developed after PCI-mediated vaccination in the 17.5 µg fimaporfin dose group. Vaccination was performed on the belly, and the picture was taken after the second vaccination, 24 h after illumination. The black circle denotes the illuminated area.

In the 50 µg dose group stopping criteria were met, as two subjects developed injection site ulceration grade 2 at one or both injection sites (three events) (**Tables 5** and **6**). The dose was therefore reduced to 25 µg, which significantly decreased the side effects. Although stopping criteria were not met, this dose was deemed as poorly tolerated with regards to pain during illumination. Two subjects at this dose were not able to receive the full light dose at one or both injection sites (one of the subjects was withdrawn from the study due to pain during illumination). Because of the pain issue with the 25 µg fimaporfin cohort, the fimaporfin dose was substantially reduced, to 2.5 µg, and a dose escalation was performed from this dose level. The 2.5 µg dose was well tolerated, as were doses of 5, 7.5 and 12.5 µg. At 17.5 µg three subjects developed local administration site reactions of erythema that were generally mild, but exceeded 10 cm (**Figure 3**), thereby meeting the dose stopping criteria.

**Table 5:** Total 14 subjects discontinued dosing either due to development of AEs or Dose stopping criteria being met in the cohort.

Fimaporfin dose	Subject	Reason for discontinued treatment
50 µg	207	Cohort discontinued
	208	Cohort discontinued
	209	Ulceration (Stopping criteria met)
	210	Cohort discontinued
	211	Cohort discontinued
	212	Ulceration (Stopping criteria met)
25 µg	215	Unable to tolerate light dose^1^
	224	Unable to tolerate light dose^2^
17.5 µg	250	Cohort discontinued
	251	Cohort discontinued
	252	Erythema (Stopping criteria met)^3^
	253	Cohort discontinued
	254	Erythema (Stopping criteria met)^3^
7.5 µg	264	Local reaction/erythema^3^

When the stopping criteria for a dose cohort was met, all subjects in that cohort discontinued further treatment. ^1^withdrawn after second ID dosing due to pain (completed 2 KLH doses but only one HPV dose), ^2^withdrawn due to pain during first dosing (physician decision), ^3^local erythema ≥10 cm in ≥2 subjects.

**Table 6:** Subject Treatment Disposition.

Fimaporfin dose	Subjects	Completed all vaccinations		Completed HPV vaccinations			Completed KLH vaccinations	
	(N=)	HPV	KLH	1^st^ dose	2^nd^ dose	3^rd^ dose	1^st^ dose	2^nd^ dose
0	12	12 (100%)	12 (100%)	12 (100%)	12 (100%)	12 (100%)	12 (100%)	12 (100%)
0.75 µg	6	6 (100%)	6 (100%)	6 (100%)	6 (100%)	6 (100%)	6 (100%)	6 (100%)
1.5 µg	6	6 (100%)	6 (100%)	6 (100%)	6 (100%)	6 (100%)	6 (100%)	6 (100%)
2.5 µg	10	10 (100%)	10 (100%)	10 (100%)	10 (100%)	10 (100%)	10 (100%)	10 (100%)
5 µg	12	12 (100%)	12 (100%)	12 (100%)	12 (100%)	12 (100%)	12 (100%)	12 (100%)
7.5 µg	12	11 (92%)	12 (100%)	12 (100%)	12 (100%)	11 (92%)	12 (100%)	12 (100%)
12.5 µg	6	6 (100%)	6 (100%)	6 (100%)	6 (100%)	6 (100%)	6 (100%)	6 (100%)
17.5 µg	6	1 (17%)	5 (83%)	6 (100%)	5 (83%)	1 (17%)	6 (100%)	5 (83%)
25 µg	12	10 (83%)	11 (92%)	11 (92%)	10 (83%)	10 (83%)	11 (92%)	11 (92%)
50 µg	6	0 (0%)	2 (33%)	6 (100%)	2 (33%)	0 (0%)	6 (100%)	2 (33%)

Subjects in the fimaporfin dose-finding part could receive up to 3 doses with HPV and 2 doses with KLH, Subjects in the highest dose groups discontinued treatment due to cohort stopping criteria being met or for safety reasons (see **Table 5**).

Although pain was generally not a problem at doses lower than 25 µg fimaporfin, pain scores during illumination were variable between subjects, and mild pain was also observed in some subjects in the lower dose groups. The pain usually vanished immediately after the end of light application.

As will also be apparent from **Figure 1**, altogether 14/88 (15.9%) subjects in the fimaporfin dose-finding part discontinued dosing; seven subjects discontinued due to AEs (all being local injection site events) and seven subjects due to their study cohort reaching predefined stopping criteria (50 and 17.5 µg dose groups) (**Tables 5** and **6**). Discontinuation AEs were ulcerations (moderate) in two subjects in the highest dose group (50 µg), mild erythema in three subjects [17.5 µg (n=2) and 7.5 µg (n=1)], and pain during light application in two subjects [not able to receive the full light dose (25 µg)]. None of the subjects in the 50 µg dose completed the full HPV or KLH vaccination regimen, and in the 17.5 µg dose group, one subject received only one HPV dose, four subjects received two doses and only one subject completed all three HPV doses. In addition, one subject in the 7.5 µg dose group did not receive the third HPV dose.

Taken together, PCI could be safely applied to intradermal vaccination in humans, with no unexpected and mostly mild systemic AEs. At higher dose levels there were some local tolerability issues, but doses below 17.5 µg fimaporfin were well tolerated and are suitable for use in later studies.

### Immune Responses to KLH and HPV16 E7 Peptide Vaccination

**Figure 4:**
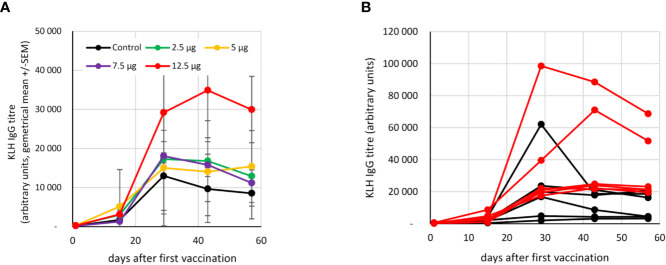
Antibody response to KLH vaccination. The presence of anti-KLH IgG was analysed by ELISA as described under *Materials and Methods*. Vaccinations were performed at days 1 and 15. **(A)** Geometrical mean (+/- SEM) of IgG titres in different fimaporfin dose groups. **(B)** IgG titres at the different time points in single study subjects in the control (black lines) and the 12.5 µg fimaporfin (red lines) groups.

The humoral response to KLH was measured in the control and 2.5-12.5 µg fimaporfin dose groups. The titer of circulating KLH-specific IgG antibodies increased in all the study subjects after the second vaccination. However, as compared to the control group, the response in vaccinated patients co-treated with PCI at a fimaporfin dose of 12.5 µg displayed a >3-fold higher KLH antibody titer at days 28 [p=0.039, t-test (unpaired, two-tailed)] and 43 (p=0.030) (**Figure 4A**). Notably, the response in the 12.5 µg fimaporfin group was more consistent with strong responses observed in all subjects, while a rather weak antibody response (< 5000 units) was observed in two of the six subjects in the control group (**Figure 4B**).

**Figure 5:**
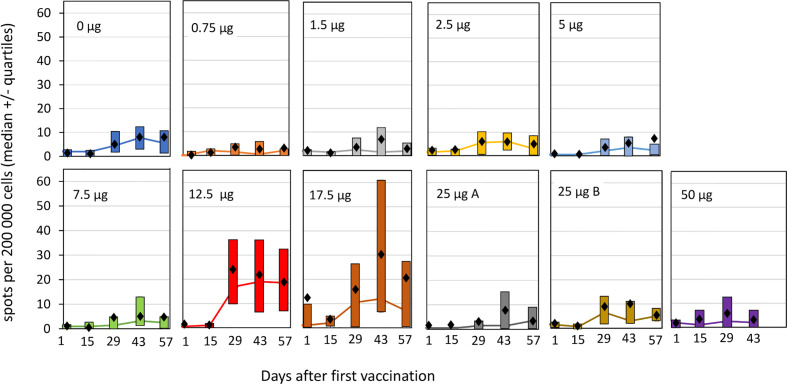
ELISpot responses against HPV16 E7 by cohorts and timepoint. PBMCs from the study subjects were isolated before and at different time points after vaccination, and subjected to ELISpot analysis after re-stimulation with the HPV E7 peptides used for the vaccination, as described under *Materials and Methods*. The spot counts for each sample were background corrected for simplified interpretation by subtracting the mean of the negative control wells from each donor from each antigen measurement from that donor. If the difference was <0, it was set to a value of 0. Each panel shows the result for an individual dose group. The lines indicate median values with the upper and lower quartiles indicated by the boxes. Mean values are shown as black diamonds. Group 25 µg A received the normal light dose of 1 J/cm^2^ at an irradiance of 5 mW/cm^2^, group 25 µg B received a light dose of 0.5 J/cm^2^ at an irradiance of 2.5 mW/cm^2^.

Antigen specific T-cells responses were analyzed by IFN-γ ELISpot as well as by multiparametric flow cytometry. Vaccination in the absence, or with low doses, of fimaporfin did not result in mean HPV-specific T-cell reactivity detectable above the LOD (15 spots per 200,000 cells, see *Materials and Methods*) in the ELISpot assay (**Figure 5**). However, a T-cell response was detected in the groups receiving co-treatment with 12.5 and 17.5 µg fimaporfin, amounting to mean spot count between 20 and 30 spots per 200,000 cells. The PCI enhanced response to HPV peptide vaccination was first observed 14 days after the second vaccination (day 29), reached a peak 2 weeks after the third vaccination (day 43), and declined at day 57 (28 days after the third vaccination). The HPV reactivity in the 17.5 µg dose group seems to taper off to a larger degree than in the 12.5 µg group, which is likely due to the missing third vaccination dose in five of six subjects the 17.5 µg fimaporfin group. In the 25 and 50 µg dose groups the ELISpot results did not seem to differ from what was observed in the control group. Thus, there seem to be a peak of immune enhancement in the 12.5–17.5 µg dose range.

**Figure 6:**
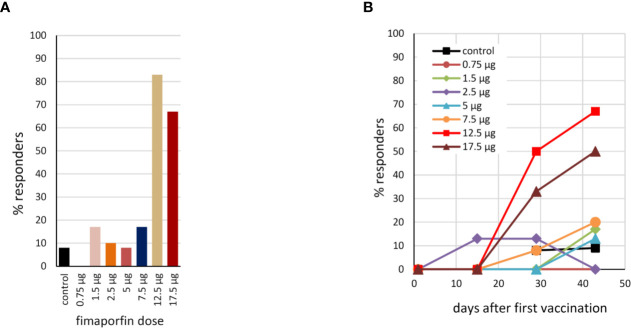
Percentage of responders to HPV16 E7 vaccination. **(A)** Based on the IFN-γ ELISpot analysis (re-stimulation with the HPV16 E7 peptides used for vaccination), the percentage of responders to the vaccination within each dosage group was calculated using as response definition: HPV response at any time after day 1; no pre-existing response. 2-fold empirical rule with median sport counts > 20 spots per 200,000 cells (see also *Materials and Methods*). **(B)** The kinetics of the response for each dose group, using the DFR(2x) response criterion (see *Materials and Methods*).

Using the response criterion defined in *Materials and Methods*, the number of responders to HPV vaccination in the different dose groups was assessed based on the ELISpot results. As can be seen from **Figure 6A**, PCI co-treatment at doses of 12.5 or 17.5 µg fimaporfin strongly increased the number of subjects responding to vaccination. While only 8% of the control subjects showed a response, the response rate in the 12.5 and 17.5 µg dose groups was 83% and 67%, respectively. **Figure 6B** shows the kinetics of the responses, indicating that a considerable number of responses were seen already after two vaccinations, but that adding a third vaccination further increased the response rate. The number of responders to vaccination with KLH was also assessed by ELISpot. There was a significant response rate in all study groups, including 50% of the control group. However, this was increased to 100% when PCI co-treatment with 12.5 and 17.5 µg fimaporfin was given (**Supplementary Figure 2**).

In order to determine the phenotype of the T-cells responding to vaccination, PBMCs from the control group, the 2.5 µg and the 12.5 µg fimaporfin dose groups were stimulated *in vitro* and re-stimulated with different HPV16 E7 peptide mixes before being evaluated by multiparametric flow cytometry The 2.5 µg fimaporfin group was selected in addition to the control group and the 12.5 µg fimaporfin group since CD8 responses have been observed already at this dose level in mouse studies (Høgset, unpublished). The functional markers analyzed were the cytokines IFN-γ, IL-2 and TNF-α as well as the activation markers CD137 and CD154. Since the cell expansion step employed in this assay introduces possibilities for skewing of the samples, we did not use this assay for performing exact quantitative comparisons of differences in response, but rather a qualitative assessment of T-cell reactivity was performed.

The flow cytometry analysis revealed HPV-specific CD4+ T-cell responses in all tested subjects, with the exception of a borderline response in one of the control subjects (#205; **Supplementary Figure 3**). Thus, with this very sensitive assay there was no obvious difference in CD4+ T-cell responses between the control and the PCI treated groups, in contrast to what was observed with the more stringent *ex vivo* ELISpot assay.

The results also indicate that a significant fraction of the CD4+ T-cells co-express multiple functional markers, and following stimulation with HPV16 E7 22-mer, the proportion of cells simultaneously expressing all four functions (CD154, IFN-γ, IL-2, and TNF-α) or the combinations of CD154, IFN-γ and TNF-α and CD154, IL-2 and TNF-α appears to be larger in the 2.5 and 12.5 µg dose groups, as compared to the control group (**Supplementary Figure 4**).

**Figure 7:**
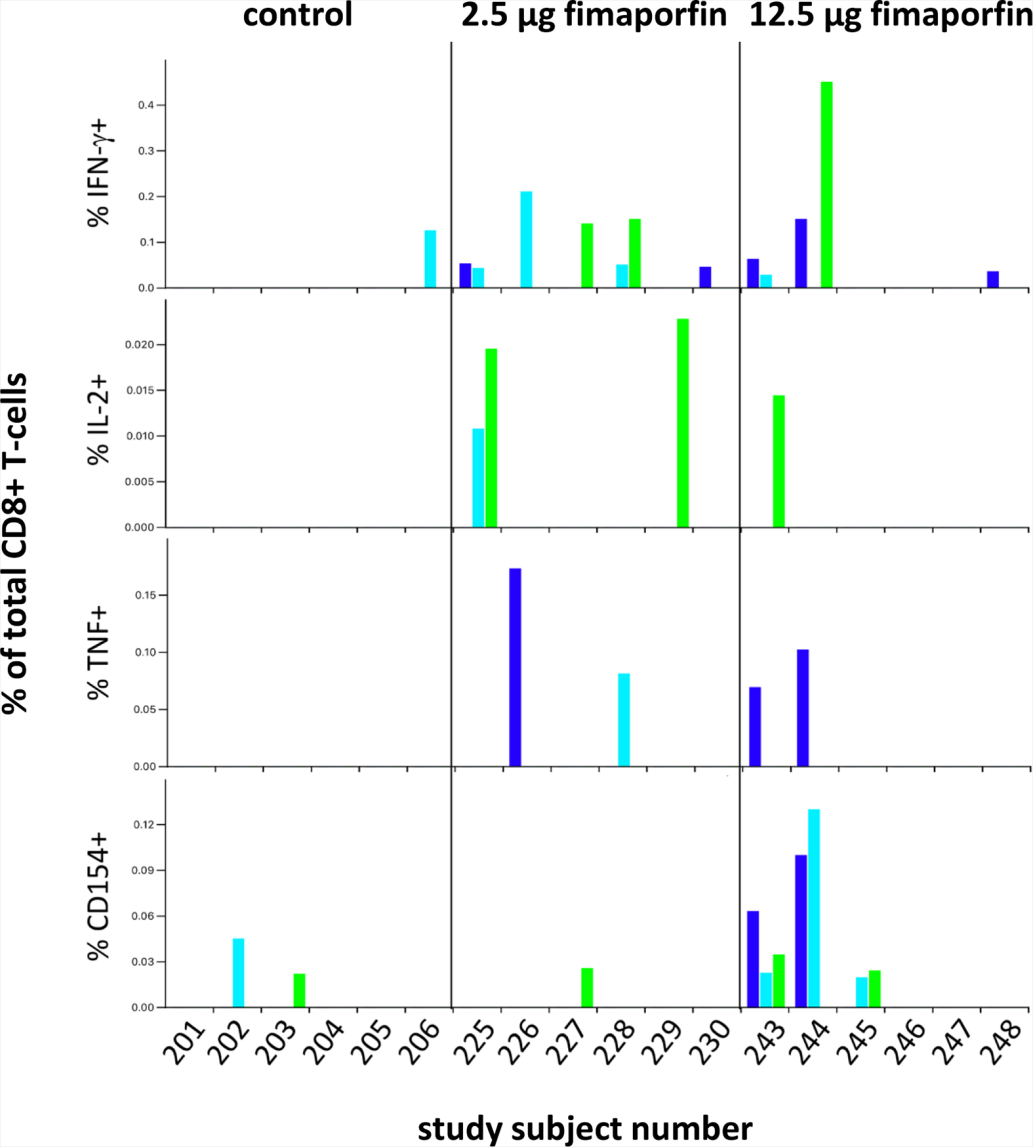
CD8+ T-cell responses to HPV16 E7 peptides. The expression of IFN-γ, IL-2, TNF-α, and CD154 was assessed in CD8+ T-cells following stimulation with HPV16 E7 22-mer peptides (described under *Materials and Methods*) for each analyzed sample. Results for the three time-points [d(ay)29, d43, d57] are indicated by the colored bars: d29 dark blue, d43 light blue, and d57 green. All measurements have been background-subtracted.

CD8+ T-cell responses to the positive control SEB were relatively consistent across time-points for individual subjects for the production of IFN-γ, TNF-α, and CD154, with the expression of IL-2 and CD137 being less consistent (**Supplementary Figure 5**). There seemed to be a lower reactivity to the SEB antigen in some of the subjects in the 12.5 µg dose group (notably subjects 244, 245, and 248). This might indicate a lower quality of the cells in these samples, but the fact that some of these samples show an HPV E7 response for functional markers (**Figure 7**) may indicate that the low response seen with the SEB positive control was due to natural individual variations in the SEB response rather than an impairment of the cell samples. A CD8+ T-cell response to the HPV16 E7 22-mer peptides at least at one time-point was detected in three of the six tested control subjects and in 6/6 and 4/6 subjects in the 2.5 µg and 12.5 µg fimaporfin groups, respectively (**Figure 7**). While no subjects in the control groups developed a response at two or more timepoints, such responses were observed for 3/6 subjects both in the 2.5 and in the 12.5 µg dose groups (**Figure 7**). Furthermore, 1/6 and 2/6 subjects exhibited HPV E7 22-mer-specific responses at all 3 time-points tested in the 2.5 and 12.5 µg dose groups, respectively (**Figure 7**).

While the cells in the control group samples produced only CD154 or IFN-γ, samples from the 2.5 µg dose group exhibited any of the four functional markers (IFN-γ, IL-2, TNF-α, CD154), with subject 225 (d43) producing both IFN-α and IL-2, and subject 228 (d43) producing both IFN-γ and TNF-α. Samples from the 12.5 µg dose groups showed production of any of the four functional markers, in some cases with co-production of IFN-γ and CD154 (243 d43), IL-2 and CD154 [subject 243 (d57)], or even IFN-γ, TNF-α, and CD154 [subject 243 (d29), subject 244 (d29)].

**Figure 8:**
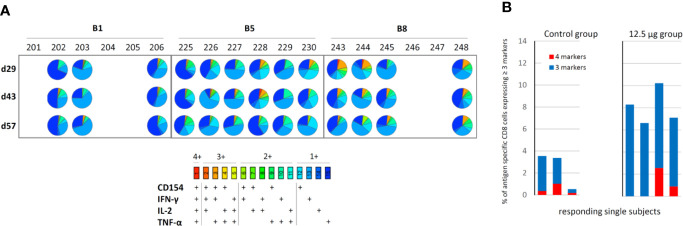
Analysis of CD8+ T-cell multifunctionality. PBMCs were incubated with pools of HPV peptides for 10 days, re-stimulated with HPV E7 22-mer peptides, stained with antibodies recognizing various surface and functional markers (CD3, CD4, CD8, IFN-γ, TNF-α, IL-2, CD154), and analysed by flow cytometry as described under *Materials and Methods*. **(A)** Assessment of the co-expression of CD154, IFN-γ, IL-2, and TNF-α. The pie charts illustrate the relative representation of cells co-expressing all four, different combinations of three, different combinations of two, or only a single of these functional markers within total functional cells (i.e. all cells that are positive for CD154 and/or IFN-γ and/or IL-2 and/or TNF), for those samples that had measurable CD8+ T-cell responses to HPV16 E7 peptide re-stimulation as defined in *Materials and Methods*. The data are not background-subtracted. **(B)** Percentage of CD8-cells expressing multiple (≥3) functional markers. Results are shown for each subject defined as a responder under the criteria described in *Materials and Methods*.

The multifunctionality of the CD8+ T-cell response to HPV E7 22-mer stimulation was further analyzed using pie charts highlighting all the possible combinations of functional markers measured. **Figure 8A** illustrates the resulting pie charts for those samples that yielded a positive response (see **Figure 7**). The **Figure 8A** pie charts appear to have a higher diversity of responses than the **Figure 7** histograms might indicate; this is due to the fact that the pie chart data is not background-subtracted. The results indicate that at least some of the cells co-express multiple functional markers, as visualized by the turquoise-to-red pie segments. Such multi-functional cells represent a larger proportion of total responding CD8+ T-cells in subjects that were treated with PCI (2.5 and 12.5 µg dose groups) than in control subjects, with the proportion of cells expressing any combination of three or four functional markers being about 4 times higher in the 12.5 µg dose group than in the control group (**Figure 8B**).

## Discussion

It has previously been shown that the PCI technology with the photosensitizing compound fimaporfin (TPCS_2a_) gives a strong enhancement of MHC class I presentation of peptide antigens in antigen presenting cells *in vitro* (4), and that this effect can be exploited to improve the CD8+ T-cell response to peptide and protein vaccination in mice (3–9). The present work represents the first study to translate these pre-clinical findings into the clinical setting, through a phase I dose-finding study using the PCI technology for vaccination with peptide and protein antigens in combination with the adjuvant poly-ICLC (Hiltonol). The primary objective of the study was to assess the safety of intradermal vaccination with PCI in combination with Hiltonol, and to define a fimaporfin dose suitable for use in later clinical studies. The results showed that the use of the PCI/Hiltonol combination in humans was safe, with generally mild adverse events localized at the site of vaccination, and identified fimaporfin doses lower than 17.5 µg as well tolerated, with higher doses being less tolerable due to local reactions. The secondary objective of the study was to assess immune responses induced by PCI-mediated vaccination, and the results indicated that the employment of PCI increased the number of subjects that exhibited a T-cell response to an HPV long peptide vaccine over what was achieved with the antigen/Hiltonol combination without PCI. Moreover, the use of PCI seemed to result in a more consistent and multifunctional CD8+ T-cell response.

It is well established that photodynamic therapy of e.g. skin cancer can induce substantial pain in a significant fraction of the patients and that high dose photochemical treatment doses can induce skin ulcers (26). Thus, in the clinical study we first conducted a safety run-in part in order to define a photochemical dose that would be tolerable as the starting dose for the main study part (fimaporfin dose-finding part). For the run-in study, a starting dose of 100 µg fimaporfin was selected, as this dose has been shown to induce immune responses in mice; and also had good tolerability in minipigs. However, in the present study, the highest doses of fimaporfin (50 and 100 µg) were not tolerated due to development of ulcers at the injection site. Ulcerations have also been reported with other vaccination technologies using adjuvants (27) and is not uncommon e.g. in a setting of therapeutic cancer vaccination. Although the local tolerability was significantly better at the 25 µg dose, it was still not deemed acceptable due to pain during illumination in some of the study subjects. The starting dose for the fimaporfin dose escalations was therefore reduced to 2.5 µg, which was well tolerated, as were doses of 5, 7.5, and 12.5 µg. Development of mild erythema was seen at 17.5 µg and dose escalation stopping criteria for the study were met as these exceeded 10 cm in longest diameter. Fimaporfin doses of 0.5 and 1.5 µg fimaporfin were also tested, and as could be expected these doses exhibited excellent tolerability, with only mild local reactions and negligible pair being observed.

Local tolerability events increasing with the fimaporfin dose levels was the only safety issue observed with the PCI vaccination regimen tested, systemic adverse reactions were generally mild and of a character that is expected in a vaccination study. Thus, PCI-mediated vaccination with fimaporfin doses below 17.5 µg can safely be further explored in humans.

As the secondary objective of the study we assessed various aspects of the immune response to PCI-mediated vaccination. Here, the most interesting finding was that PCI vaccination with a fimaporfin dose of 12.5 µg led to an almost 10-fold increase in the percentage of study subjects exhibiting a T-cell response to HPV16 E7 peptide vaccination, as compared to a control group receiving the same vaccine without PCI. A positive effect was seen also in the 17.5 µg group despite that only 1/6 subjects in this group received the full HPV peptide vaccination schedule.

It is known that HPV E7 synthetic long peptide vaccination leads to strong CD4+ T-cell responses, but that with such antigens it is much more difficult to induce CD8+ T-cell responses (25, 28, 29). Thus, the enhanced overall T-cell response (IFN-γ ELISpot) observed in the 12.5 and 17.5 µg fimaporfin groups probably to a large degree represents an effect on the magnitude of the HPV-specific CD4+ type 1 T-cell response. The occurrence of CD4+ T-cell responses were also corroborated by the more sensitive, but less stringent, flow cytometry analysis, where most subjects in the analyzed groups (except one subject in the control group) exhibited strong CD4+ T-cell responses. Increased antigen-specific CD4+ T-cell responses after PCI treatment have also been observed in pre-clinical studies with protein antigens (Selbo *et al*., manuscript in preparation).

In the present work also a significant increase in the IgG antibody response to the KLH protein antigen was observed. A similar effect has been observed in several pre-clinical studies with protein and long peptide antigens used in combination with poly(IC) (Selbo *et al*., manuscript in preparation). The mechanism behind the stimulation of antibody responses may include an enhancement of CD4+ T-cell responses, potentially stimulating B-cells to produce antibodies (30).

The main cellular effect of the PCI technology is inducing endosomal permeabilization, among other things leading to enhanced MHC class I antigen presentation (4, 5). Given that endocytic vesicles are important in the MHC class II presentation pathway, it may seem counter-intuitive that PCI should enhance CD4+ T-cell responses. There may however be several explanations for this. Firstly, during illumination there will be a light dose gradient downwards and sideways in the tissue. This means that APCs located at different locations in the tissue will receive different “doses of PCI”. Thus, the situation in the vaccination area may well be that some APCs (near to the light source) will receive a high “PCI dose” permeabilizing all endocytic vesicles and leading mainly to MHC class I presentation in these cells (maybe at the expense of class II presentation). Other cells will receive an intermediate “PCI dose” giving both MHC class I and II presentation in the same cell, and some cells located “far away” from the injection/illumination site (e.g. deeper in the tissue), may receive antigen, adjuvant and fimaporfin but only a small light dose, meaning that the “default” MHC class II presentation will dominate in these cells. Furthermore, PCI-induced stimulation of CD4+ T-cell responses may occur through several mechanisms. i) The photochemical treatment in itself can have a general immunostimulatory effect e.g. by the induction of cytokine production [e.g. IL-6 (5);] and by the upregulation of activation markers on APCs (4). ii) A substantial fraction of MHC class II presentation may take place by pathways involving antigen localization in the cytosol (31), and presentation mediated by such pathways may be enhanced by PCI, because of increased availability of antigens in the cytosol. iii) Various forms of photochemical treatments have been shown to induce autophagy (32), and autophagy is known to be involved in some types of MHC class II antigen presentation (33, 34).

Taken together, the aggregated effects, measurable in blood samples, of these possible different processes may lead to a stimulation both of CD8+ T-cell, CD4+ T-cell and antibody responses. In most vaccination settings stimulation of all these branches of the immune system would be highly advantageous.

In contrast to the finding of an enhanced antibody response observed in the present study, earlier studies on PCI-mediated immunization with adjuvant-free particle- (6) or liposome- (7) based vaccines have indicated that PCI may have a negative effect on humoral immune responses to the ovalbumin (OVA) antigen, and also that that the effect of PCI on CD8+ T-cell responses is independent of CD4+ T-cells (9). The reason for the seemingly different effects of PCI on antibody production has so far not been elucidated, but one possibility is that it may be related to the use of poly(IC) based adjuvants in the studies showing av positive effect of PCI on antibody production.

Earlier clinical experience with HPV16 E7 peptide vaccination have shown that it is difficult to induce CD8+ T-cell responses to HPV16 E7 peptides in humans, with response rates of 11% (25) and 50% (28) being reported using vaccines containing nine HPV16 E6 plus four HPV16 E7 peptides. Thus, as discussed above it was expected that the IFN-γ response detected by ELISpot analysis in the present study using only two HPV16 E7 peptides was mostly contributed by CD4+ T-cells. Therefore, the flow cytometry analysis was employed to explore the effect of the PCI treatment specifically on CD8+ T-cell responses. Samples from three selected study groups were selected for this analysis, which was performed using the same methodology as used in the above cited studies (25, 28). As could be expected from previous studies with HPV E7 peptides (24, 27) in general the CD8+ T-cells responses observed in the present study were weak (<0.5% positive cells), while the CD4+ T-cell responses seemed much more robust, with up to 15% positive cells in same samples. The flow cytometry analyses revealed HPV-specific CD8+ T-cell responses in 50% of the subjects in the control group, however, such responses were likely to be weak since they could be detected at only one time point. In comparison, for the CD8+ T-cell responses the groups of subjects co-treated with fimaporfin had a tendency of a higher overall response rate, and exhibited more robust responses, with responses seen at several time points and to more than one of the analyzed markers. Apparently, these responses were of better quality than the responses in the control group, as reflected in the multifunctionality of the responses measured. A similar effect on functionality has been observed also in several animal studies (Selbo *et al*., manuscript in preparation). Given the importance of the functionality of the CD8+ T-cells for effective immune responses to tumors and viral infections (35–37) this finding is very encouraging for the future exploration of PCI-mediated vaccination in therapeutic vaccination settings.

In conclusion, this clinical study demonstrates that the use of PCI in combination with a poly(IC) based adjuvant is safe and can enhance both cellular and humoral immune responses to ID vaccination over what is achieved with a vaccine given without PCI. As shown in animal studies the properties of the PCI technology may be especially useful for enhancing the effect of polypeptide based therapeutic cancer vaccines (Selbo *et al*., manuscript in preparation), and based on the positive clinical results in the present study, this will be explored in further clinical studies.

## Data Availability Statement

The raw data supporting the conclusions of this article will be made available by the authors, without undue reservation.

## Ethics Statement

The studies involving human participants were reviewed and approved by North East–York Research Ethics Committee, UK (16/NE/0198). The patients/participants provided their written informed consent to participate in this study.

## Author Contributions

TO and AH contributed to conception and design of the clinical study including immune monitoring analysis plan, immune data interpretation, wrote the first draft of the manuscript and final manuscript. TO was the project manager for the clinical study. SJ contributed to ELISpot assay design, ELISpot data analysis and interpretation, flow cytometry analysis and data interpretation as well as to the manuscript. MW contributed to flow cytometry analysis and data interpretation as well as to the manuscript. MH, AN, and VE contributed to data acquisition by ELISpot analysis. SBo and NL contributed to data acquisition by flow cytometry and analysis. PS provided scientific input and contributed to manuscript writing and final approval of manuscript. SvdB contributed to data interpretation and to the manuscript. HO contributed to design of the clinical study and safety monitoring. All authors contributed to the article and approved the submitted version.

## Funding

The study was funded by PCI Biotech AS. PCI Biotech has received funding from the Norwegian Research Council for a Project (Project no. 269817) including the present study.

## Conflict of Interest

TO and AH are employees of PCI Biotech AS and own shares and share options in the Company. AH and PS are inventors on several patents and patent Applications on the PCI Technology. HO and SJ work as consultants for PCI Biotech, and SJ is working for ZellNet Consulting, Inc. VE is an employee of PCI Biotech.

The remaining authors declare that the research was conducted in the absence of any commercial or financial relationships that could be construed as a potential conflict of interest.
