# Supplementary material for: Photochemical Internalization Enhanced Vaccination Is Safe, and Gives Promising Cellular Immune Responses to an HPV Peptide-Based Vaccine in a Phase I Clinical Study in Healthy Volunteers
Source: Front Immunol. 2021 Jan 8;11:576756. doi: 10.3389/fimmu.2020.576756 (PMC7819858; doi:10.3389/fimmu.2020.576756)
Supplement: Supplementary file 1 [file DataSheet_1.pdf]

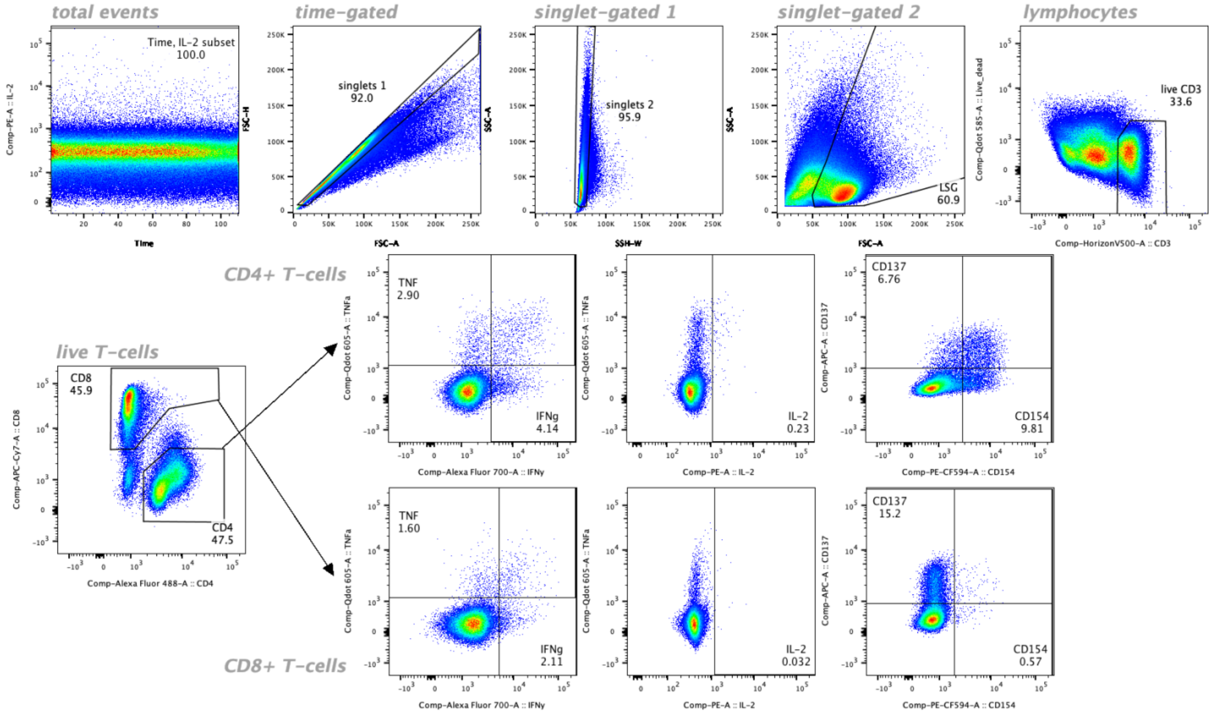

Supplementary Figure 1. Gating tree for functional evaluation of T-cells. After excluding any acquisition segments impacted by potential mini-clogs or air bubbles (time vs. IL-2PE), singlets were selected using two consecutive gates (FSC-A vs. FSC-H, and SSC-W vs. SSC-A). Lymphocytes (FSC-A vs. SSC-A), then live T-cells (CD3H500 vs. live/deadV585) were then identified. Within these, CD4+ and CD8+ T-cells were selected (CD4Ax488 vs. CD8APC-Cy7) and the expression of functional markers investigated (IFN-γ-Ax700 vs. TNFQD605; IL-2PE vs. TNFQD605; CD154PE-CF594 vs. CD137APC). Shown are dot plots for SEB-stimulated d29 PBMC from donor 202.

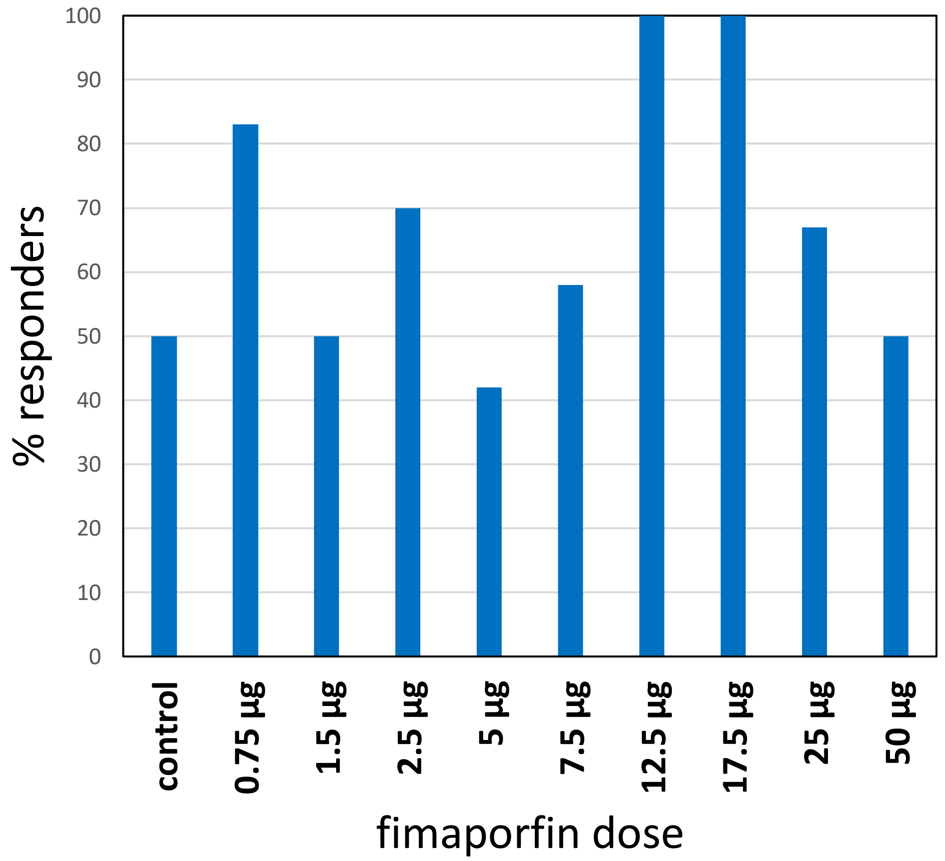

Supplementary Figure 2. Percentage of responders to KLH vaccination. Based on the IFN-γ ELISpot analysis the percentage of responders to the KLH vaccination within each dose group was calculated using as response definition: HPV response at any time after Day 1; no pre-existing response. 2-fold empirical rule with median sport counts > 20 spots per 200,000 cells (see also Materials and Methods).

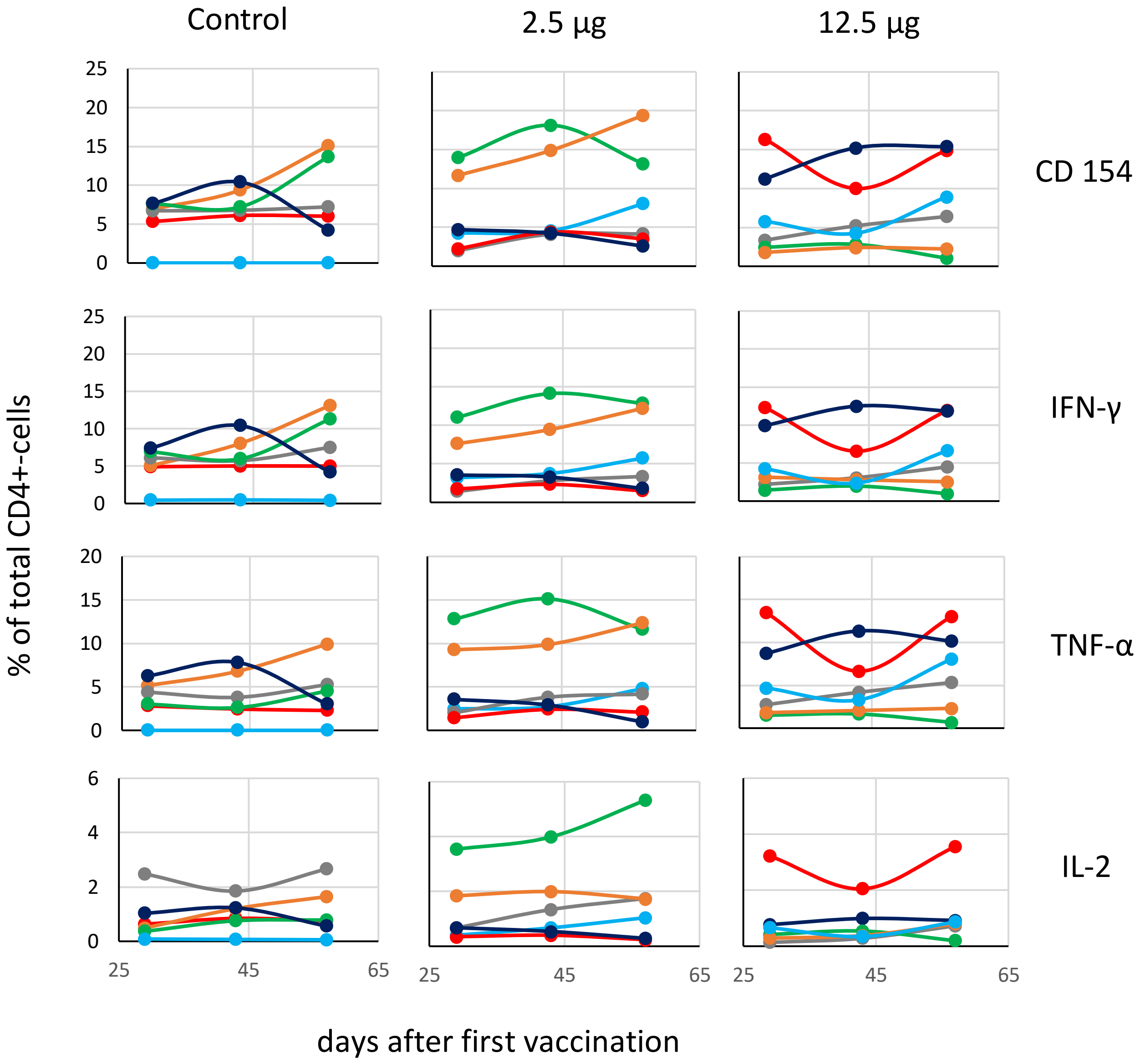

Supplementary Figure 3. Flow cytometry analysis of CD4+ T-cell responses to HPV16 E7 peptides. PBMCs were incubated with pools of HPV peptides for 10 days, re-stimulated with HPV E7 22-mer peptides, stained with antibodies recognizing various surface and functional markers (CD3, CD4, CD8, IFN-γ, TNF-α, IL-2, CD154), and analysed by flow cytometry as described under Materials and Methods.. Results in CD4+-positive cells at the time-points (d29, d43, d57) are shown for CD154, IFN-γ, TNF-α and IL-2 from single subjects in these groups. Each study subject is shown in the same color within each dose group.

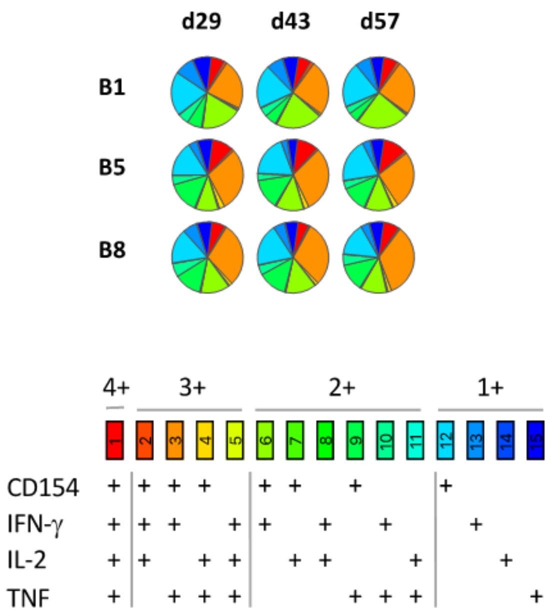

Supplementary Figure 4. Multi-functionality of CD4+ T-cell response. The co-expression of CD154 (CD40L), IFN-γ, IL-2, and TNF-α was assessed in CD4+ T-cells following stimulation with, HPV16 E7 22mer. Pie charts illustrating the relative representation of cells co-expressing all four, different combinations of three, different combinations of two, or only a single of those functional markers within total functional cells (i.e. all cells that are positive for CD154 and/or IFN-γ and/or IL-2 and/or TNF-α) are shown. Each pie represents the average of all patients measured for a given group and time-point combination. These data are not background-subtracted.

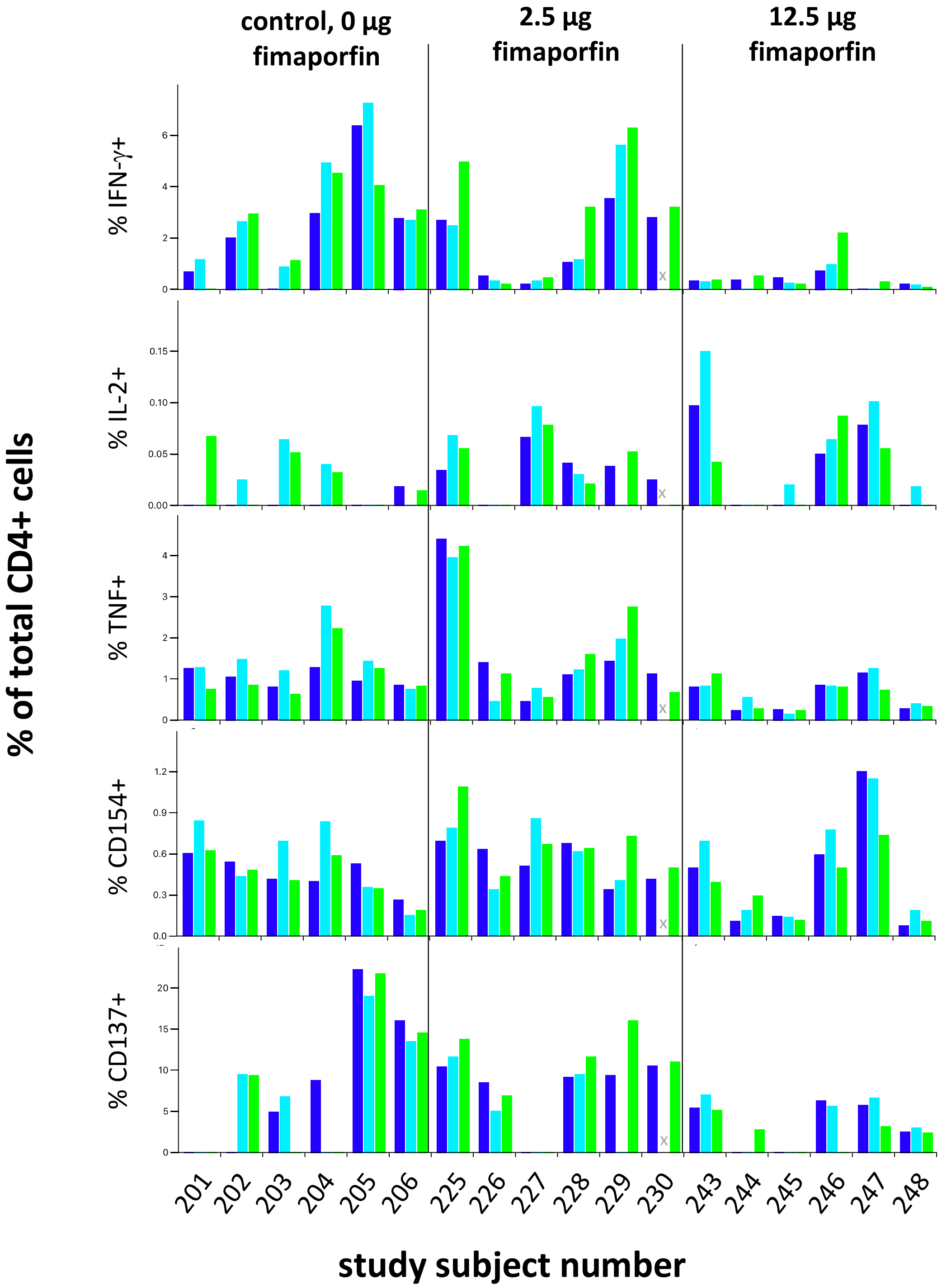

Supplementary Figure 5. CD8+ T-cell responses to SEB. The expression of IFN-γ, IL-2, TNF-α, CD154 (CD40L), and CD137 (4-1BB) was assessed in CD8+ T-cells following stimulation with SEB for each analyzed sample. Results for the three time-points (d29, d43, d57) are indicated by the colored bars: d29 dark blue, d43 light blue and d57 green. All measurements have been background-subtracted.
